# The Effect of Atmospheric Cold Plasma on Bacterial Stress Responses and Virulence Using *Listeria monocytogenes* Knockout Mutants

**DOI:** 10.3389/fmicb.2019.02841

**Published:** 2019-12-11

**Authors:** Apurva Patange, Conor O’Byrne, Daniela Boehm, P. J. Cullen, Kevin Keener, Paula Bourke

**Affiliations:** ^1^School of Food Science and Environmental Health, Technological University Dublin, Dublin, Ireland; ^2^Bacterial Stress Response Group, School of Natural Sciences, National University of Ireland, Galway, Galway, Ireland; ^3^School of Chemical and Biomolecular Engineering, The University of Sydney, Sydney, NSW, Australia; ^4^Department of Food Science and Human Nutrition, Iowa State University, Ames, IA, United States; ^5^School of Biological Sciences, IGFS, Queen’s University Belfast, Belfast, United Kingdom

**Keywords:** *Listeria monocytogenes*, stress genes, atmospheric cold plasma, gene expression, biofilm, sub-lethal stress

## Abstract

*Listeria monocytogenes* is an opportunistic intracellular pathogen commonly associated with serious infections and multiple food-borne outbreaks. In this study, we investigated the influence of atmospheric cold plasma (80 kV, 50 Hz) on *L. monocytogenes* (EGD-e) and its knockout mutants of *sigB*, *rsbR*, *prfA*, *gadD*, and *lmo0799* genes at different treatment time intervals. Further, to ascertain if sub-lethal environmental stress conditions could influence *L. monocytogenes* survival and growth responses, atmospheric cold plasma (ACP) resistance was evaluated for the cultures exposed to cold (4°C) or acid (pH 4) stress for 1 h. The results demonstrate that both wild-type and knockout mutants were similarly affected after 1 min exposure to ACP (*p* > 0.05), with a difference in response noted only after 3 min of treatment. While all *L. monocytogenes* strains exposed to acid/cold stress were hypersensitive to ACP treatment and were significantly reduced or inactivated within 1 min of treatment (*p* < 0.05). The results indicate *sigB* and *prfA* are important for general stress resistance and biofilm, respectively, loss of these two genes significantly reduced bacterial resistance to ACP treatment. In addition, exposure to sub-lethal 1min ACP increased the gene expression of stress associated genes. *SigB* showed the highest gene expression, increasing by 15.60 fold, followed by *gadD2* (7.19) and *lmo0799* (8.6) after 1 min exposure. Overall, an increase in gene expression was seen in all stress associated genes analyzed both at 1 min treatment; while long treatment time reduced the gene expression and some cases down-regulated *prfA* and *gadD3* gene expression. By comparing the response of mutants under ACP exposure to key processing parameters, the experimental results presented here provide a baseline for understanding the bacterial genetic response and resistance to cold plasma stress and offers promising insights for optimizing ACP applications.

## Introduction

*Listeria monocytogenes* 1/2a serotypes are persistent strains, which are commonly associated with multiple food-borne outbreaks. Listeriosis is a relatively rare disease, however, there was a significant increase observed in 2008–2015. In 2015, the EU reported 2,206 numbers of confirmed cases of Listeriosis in humans and approximately 964 hospitalizations (European Food Safety Authority [EFSA], 2017). The main vehicle for transmission of *L. monocytogenes* to humans is through consumption of contaminated food products. Because of the theoretical risks and actual outbreaks associated with *L. monocytogenes*, it is important to understand how they respond and or survive on fresh produce in response to novel decontamination processes. The ability of the organism to persist and thrive under broad range of environmental conditions is attributable to the capacity to respond efficiently to environmental stresses. Many of the foodborne pathogens including *L. monocytogenes* possess adaptive responses to physiological environmental stresses such as acid, heat, salt, alkali and oxidative, which are also likely exposure conditions in the food processing environment (Bucur et al., 2018).

In *L. monocytogenes* and *Bacillus subtilis*, Sigma factor B (σ^B^) was shown to have a role in growth and survival under several stress conditions (Wiedmann et al., 1998). σ^B^ of *L. monocytogenes* regulates the expression of numerous genes under environmental stress conditions. Sigma factor in association with core RNA polymerase provides a mechanism for cellular responses that are mediated through redirection of transcription initiation (Kazmierczak et al., 2005). The *L. monocytogenes*σ^B^ regulon includes more than 140 genes that are both directly and positively regulated by σ^B^, including genes encoding proteins responsible for stress response, virulence, transcriptional regulation, carbohydrate metabolism, and transport (Raengpradub et al., 2008). Furthermore, σ^B^ contributes to the ability of stationary-phase *L. monocytogenes* cells to adapt and resume growth after exposure to sub-lethal stress conditions (Ferreira et al., 2001). These exposures to sub-lethal stress may lead to enhanced survival, resistance, virulence and even cross-protection against multiple further stresses (Dhowlaghar et al., 2018). Despite the well-established role of σ^B^ allowing *L. monocytogenes* to grow under varied conditions, the studies addressing the effects of *sigB* deletion on characteristics of *L. monocytogenes* at stress conditions provide conflicting results.

Therefore, the focus of our study was to better understand the stress adaptive responses in *L. monocytogenes* and to investigate the safe use of atmospheric cold plasma (ACP) as a decontamination technology to control *Listeria* in the fresh produce food chain. Cold plasma is an ionized gas composed of ions, electrons, free radicals and (positively and negatively) charged particles. ACP treatment generates several reactive short and long-lived species including reactive oxygen species (ROS), reactive nitrogen species (RNS) which have been shown to play an important role in biological effects (Guo et al., 2015). The ROS generated have a strong oxidative effect on cell and intracellular components which can lead to lipid peroxidation, DNA and protein damage. Previously Han et al. (2016a), demonstrated cell leakage and DNA damage in ACP treated *E. coli* cells suggesting the importance of cellular regulatory and repair systems under plasma stress. Differential expression conducted on plasma treated *E. coli* K-12 cells showed higher expression of SOS regulon, oxidative associated genes and DNA repair damage genes (Sharma et al., 2009). ACP has been reported as effective for disinfection of a wide range of microbial population from different surfaces. The role of cold plasma from air at or near atmospheric pressure and room temperature has been extensively studied and has exhibited excellent antibacterial efficacy against target food pathogens, their spores and biofilms (Bourke et al., 2017). Further, recent research has also demonstrated effective application of cold plasma for toxin elimination, pesticide degradation, food and package functionalization (Cullen et al., 2017). In-package ACP treatment along with contained post treatment retention time of plasma reactive species enhanced the bacterial inactivation efficacy for decontamination of fresh produce (Ziuzina et al., 2014). However, how different microorganisms respond to plasma treatment following pre-exposure to sub-lethal levels of stress is unaddressed. Stress conditions can provoke a variety of specific and highly regulated adaptive responses, which could not only protect the bacteria from the stress, but also promote antimicrobial susceptibility (Poole, 2012). Therefore, it is important to investigate the cold plasma treatments and their influence on bacterial adaptive stress responses and resistance. To study this, the role of eight genes (*sigB, rsbR, gadD1, gadD2, gadD3, gadD2D3, lmo0799*, and *lmo0799-C56A* – Table 1) associated with stress regulation were evaluated with respect to their stress responses, plasma inactivation, response to RONS intensity and gene expression level post plasma treatment.

**TABLE 1:** Strains and mutants used in this study.

**Collection no.**	**Microorganism**	**Description**	**References**
***Listeria monocytogenes* strains**			
COB 261	EGD-e (wild-type)	Serotype 1/2a	
COB 262	EGD-e △*sigB*	Plays an important role under multiple stress conditions (heat, high osmolarity, high ethanol concentrations, high and low pH, and oxidizing agents)	Liu et al., 2019; Utratna et al., 2011
COB 455 COB 456 COB 497 COB 612	EGD-e △*gadD1* EGD-e △*gadD2* EGD-e △*gadD3* EGD-e △gadD2D3	Encodes a glutamate decarboxylase important for acid tolerance and play significant role in overall virulence of *L. monocytogenes*	Feehily et al., 2014
COB 677	EGD-e Δ*prfA*	Listeriolysin-positive regulatory factor A Virulence Regulator factor, influences expression of several virulence factors such as biofilm formation	Lemon et al., 2010
COB 672	EGD-e Δ*rsbR*	Stress regulator (positive regulator for sigma B activity)	Donaldson et al., 2009
COB644 COB610	△*lmo0799* △*lmo0799*-C56A	Sulfate Transporter and Anti-Sigma factor antagonist domain of the “stressosome” complex proteins *Rsb*S and *RsbR*, regulators of the bacterial stress activated alternative sigma factor sigma-B by phosphorylation; △*lmo0799* C56A: Cys is replaced by Ala	O’Donoghue et al., 2016

## Materials and Methods

### Bacteria Strains and Growth Conditions

*Listeria monocytogenes* EGD-e (WT) and its nine mutants (Δ*sigB*, Δ*gadD1*, Δ*gadD2*, Δ*gadD3*, Δ*gadD2D3*, Δ*prfA*, Δ*rsbR*, Δ*lmo0799*, and Δ*lmo0799-C56A*) used in this study are described in Table 1. All strains were obtained from the Department of Microbiology, NUI Galway. Strains were stored in the form of protective beads at −70°C. The stock cultures were streaked on brain heart infusion agar (BHIA) and incubated at 37°C for 24 h prior storage at 4°C.

### Media and Growth Conditions

The wild type and mutant cells were grown in sterile brain heart infusion broth (BHIB, Thermo Fisher Scientific, Ireland) for 16–18 h at 37°C. The overnight cultures were washed thrice with phosphate buffer solution (PBS, Oxoid Ltd., United Kingdom) by centrifugation at 8720*g* for 5 min and adjusted to 0.5 McFarland standards (BioMerieux, Marcy-l’Etoile, France). Final cell suspensions with concentration of 1 × 10^8^ CFU/ml were prepared in PBS (planktonic) or BHIB (biofilm).

### Resistance to Acid and Cold

To obtain mild stress-habituated cells, two stress conditions were imposed i.e., acid and cold stress. The cells were prepared by suspending the washed wild type and mutant cells either in PBS and stored in 4°C for 1 h or in acidified PBS (pH 4.0 using acetic acid) and then incubated for 1 h at 37°C. Cells that were not treated with acid/cold (in PBS alone) served as negative control. Cells prepared under this range of conditions were dispensed in 96-well plates and were then treated with ACP for 1 min with 1 h post treatment storage time (PTST).

### Biofilm Assay

Surface adhered biofilm formation was assayed in 96-well microtiter plates (Sarstedt, Nümbrecht, Germany) by using plate count and crystal violet assay as described by Jackson et al. (2002). Briefly, the bacterial suspension (200 μl) in BHIB at a concentration of 1.0 × 10^7–8^ CFU/ml was dispensed into each well of a microtiter plate. The plates were incubated at 37°C for 48 h and supernatant was changed after 24 h of incubation. To quantify biofilm formation, the planktonic cells along with non-adherent cells were removed and the wells were washed with 200 μl of sterile PBS three times. Prior to each experiment, biofilms were air dried for 40–50 min. The biofilms were then quantified by crystal violet assay as described in Stepanović et al. (2000). The absorbance was measured at 590 nm using a micro-titer plate reader (Synergy HT, Biotek Instruments Inc., Winooski, VT, United States). Each biofilm well absorbance value was corrected by subtracting the means of absorbance of a blank (un-inoculated) BHIB. The antibacterial effect of applied ACP treatment on bacterial biofilm was quantified using Plate count (PC) and XTT (2,3-bis (2-methoxy-4-nitro-5-sulfophenyl) [phenyl-amino) car-bonyl]-2H-tetrazolium hydroxide); Sigma Aldrich, Ireland) assay as previously described by Peeters et al. (2008).

### ACP Treatment Design

**FIGURE 1:**
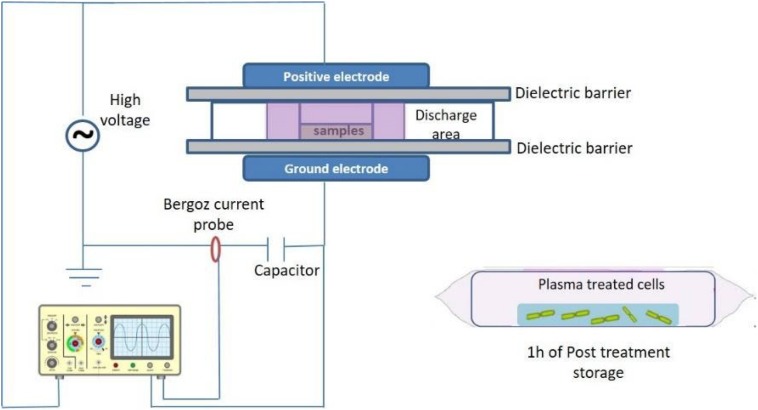
Schematic of experimental setup using DBD-ACP, DIT120 Plasma Device.

Cold plasma treatment was performed using a custom built dielectric barrier discharge (DBD-DIT120) ACP system, which is fully described and characterized in Patil et al. (2014) and Moiseev et al. (2014). ACP discharge was generated between two aluminum electrodes (15 cm diameter) separated by two polypropylene sheets in the middle, which is used as a dielectric barrier and holder for the sample container and cell samples (Figure 1). Both stressed and non-stressed bacterial cells were suspended in 96-well microtiter plates and placed inside a polypropylene container and sealed using a polypropylene bag (Cryovac, B2630, United States). The sample was placed directly in the discharge area between the two electrodes. Bacterial samples were treated for 1–5 min at 80 kV using atmospheric air as working gas followed by 1 h of PTST. The treated and non-treated samples were spread plated (volume of 0.1 and 1 ml of diluted or undiluted sample) in duplicates onto tryptic soy agar (TSA, Biokar Diagnostics, Allonne, France) agar plates and incubated at 37°C for 24–48 h. Colonies were enumerated and reported as Log_10_ CFU/ml. Experiments were performed in duplicate and replicated twice.

### Detection of Reactive Oxygen Species Post Treatment

Measurement of reactive oxygen species (ROS) generated in ACP treated bacterial suspension was performed utilizing 2′, 7′-dichlorodihydrofluorescein diacetate (DCFH-DA), a dye that is oxidized to a fluorescent dichlorofluorescein. The treated bacterial cells were incubated with DCFH-DA dye (Sigma-Aldrich, Arklow, Ireland) at the final concentration of 5 μM in PBS for 15 min at 37°C under dark conditions. After incubation, aliquots of each sample was transferred to 96-well plates and the fluorescence was measured on Synergy^TM^ HT Multi-Mode Microplate Reader (BioTek Instruments Inc., Winooski, VT, United States) at excitation and emission wavelengths of 485 and 525 nm. The data generated represent the ROS concentrations generated after ACP treatment expressed as arbitrary fluorescence units (AFU).

### RNA Isolation and RT-PCR

Transcription analysis was performed for EGD-e wild type strain. Relative expression of stress associated genes (*prfA*, *sigB*, *rsbR*, *gadD*, *lmo0799*) following exposure to ACP treatment was quantified using real-time reverse transcription-PCR (RT-PCR). RNA was isolated from ACP treated (1 and 3 min, 1 h of post treatment storage time) and untreated cells. Prior to RNA isolation, 1 ml of bacterial culture was mixed with two volumes of RNAlater^TM^ Stabilization Solution (Ambion, Austin, TX, United States). Total RNA was isolated using the RNeasy Mini Kit (Qiagen, United Kingdom) as per manufacturer’s instructions followed by DNase treatment using TURBO^TM^ DNase (Ambion, Austin, TX, United States) to remove residual genomic DNA. The quantity and quality of the isolated RNA was determined by measuring absorbance at 230, 260, and 280 nm by μDrop Multiskan^TM^ FC Microplate Photometer (Thermo Fisher Scientific, Ireland). The integrity of the purified RNA was determined using 1.3% agarose gel (containing 1.8 ml formaldehyde and 5 μl SYBR safe) and subjected to electrophoresis at 70 kV for 40 min. RNA samples were stored at −80°C until needed.

Subsequently, RNA was converted to cDNA with the use of random hexamers and SuperScript III Reverse Transcriptase kit (Invitrogen, Carlsbad, CA, United States) according to the manufacturer’s instructions. As a negative control similar amount of total RNA was subjected to cDNA synthesis reaction without the reverse transcriptase enzyme. This provides a way to assess the potential DNA contamination of each sample during the real time PCR assay. RT-PCRs (Singleplex assay) were performed using PowerUp^TM^ SYBR^®^ Green Master Mix (Applied Biosystems, CA, United States) and Applied Biosystems 7500 Fast system. The PCR was programed starting with an initial activation step at 95°C for 2 min, followed by amplification for 40 cycles at 95°C for 15 s, 30 s at various annealing temperatures, depending on the melting temperature of the set of primers (Supplementary Table S1), and 72°C for 11 s. The specificity of amplification for each product was determined by a melting curve analysis at 95°C for 5 s and 60°C for 15 s, followed by a progressive increase of the temperature to 95°C with a ramp rate of 0.11°C s^–1^, with continued measurement of fluorescence, and finally cooling of the plate at 40°C for 30 s. The housekeeping gene 16S rRNA served as an internal standard. All transcription analyses were done at least twice with two technical replicates each. Alongside each real-time PCR assay, a control reaction without added cDNA was run as a negative control. Expression of target genes (*prfA, sigB, rsbR, gadD, lmo0799*) in the treatment group relative to that in control group, normalized to 16S rRNA housekeeping gene, were calculated using delta-delta CT (2^–Δ^ ^Δ^ ^Ct^) method. Calculations were carried out following the advanced relative quantification settings of the Applied biosystem fast 7500 software program. Relative expression of each gene was calculated by comparison of its expression relative to that of the 16S rRNA gene. Results were expressed as Log_2_ Fold (2^–Δ^ ^Δ^ ^CT^); values > 1 and < 1 indicated the overexpression and the under-expression of target genes upon ACP exposure, respectively. Amplification efficiency (ε = 10^(–1/slope)^ –1) was calculated for both housekeeping and target gene from the slope of the log-linear portion of the calibration curve with four serial dilutions of *L. monocytogenes* EGD-e strain cDNA.

### Statistical Analysis

Statistical analysis was performed using SPSS 22.0 (SPSS Inc., Chicago, United States). All microbial data were pooled and average values and standard deviations determined. Statistical data analysis for intracellular ROS assay between control and ACP treated samples of wild type and mutants were performed by using analysis of variance (two-way ANOVA along with Tukey *post hoc* test). Comparisons between (i) stress (acid/cold) exposed cells and their controls and (ii) the wild type strain and its isogenic mutants were made at ACP treatment time = 0, 3, and 5 min using two-way ANOVA-Tukey *post hoc* test. For RT-qPCR data, fold change was log-transformed. To determine whether individual gene expression levels were increased/decreased after exposure to ACP at each time point, means was used to perform a one-way ANOVA, with Fisher’s Least Significant-corrected *p*-values and an α = 0.05 as the cut off for significance. Error bars indicating standard deviations from the means are displayed on graphs and tables.

## Results

### Detection of Intracellular ROS in *L. monocytogenes* After Cold Plasma Treatment

**FIGURE 2:**
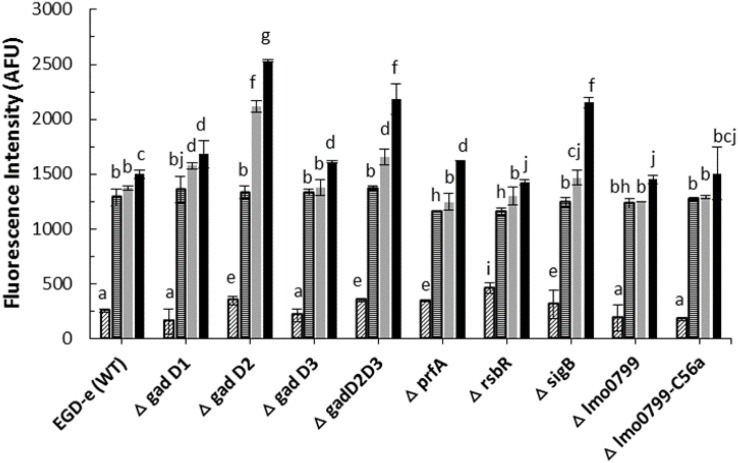
*Listeria monocytogenes* mutants and parent strain ROS density assay by DCFH. 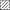, control; 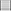, 1 min ACP treated; 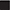, 3 min ACP treated; 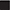 5 min ACP treated. Experimental conditions: 1, 3, 5 min treatment at 80 kV, following 1 h post treatment storage. Untreated controls followed the same preparation procedure at all post-treatment storage times. Higher fluorescence signals detected in AFU indicates higher intracellular ROS concentration as measured by DCFH-DA. Column with different letters indicate a significant difference between bacterial strains and treatment time (*p* < 0.05).

Several studies have reported that biological effects of cold plasma are mainly due to generation of reactive species (Barekzi et al., 2012; Graves, 2012). Amongst them, ROS play an important role in the bactericidal activity. Parameters such as the plasma source, gas mixture, method of treatment, composition of the substrate medium treated affect ROS generation and plasma performance (Joshi et al., 2011). In an attempt to evaluate treatment parameters influencing intracellular ROS generation, *L. monocytogenes* strains were exposed to cold plasma treatment for 1–5 min. As observed in Figure 2, 1 min cold plasma treatment significantly increased the ROS levels in both wild-type and mutant strains. The ROS levels increased in tandem with increasing treatment times in samples with post-treatment storage time of 1 h. Δ*gadD2* with 1 min treatment had a much higher signal than all other mutant strains (*p* < 0.05). After 5 min treatment, higher ROS levels were observed from Δ*gadD2*, Δ*gadD2D3*, and Δ*sigB* than wild type strain and other mutants.

### ACP Inactivation Efficacy Associated With Treatment Parameters

**FIGURE 3:**
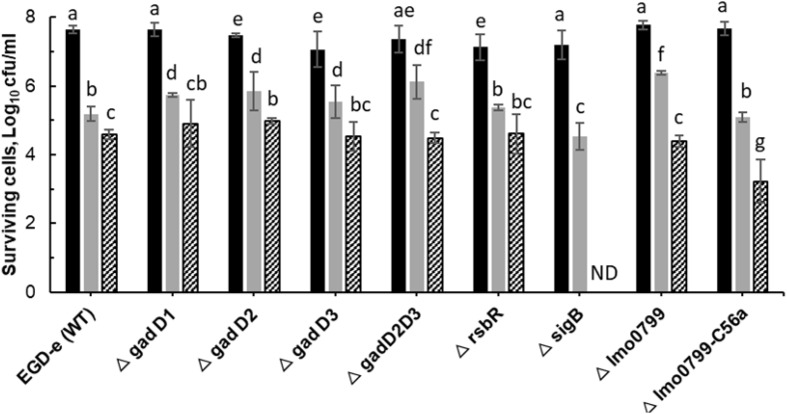
Effect of ACP on *L. monocytogenes* EGD-e wild type and its knockout mutant strains. The graph displays surviving population of *L. monocytogenes* after ACP treatment at variable time range (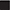, untreated control; 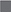, 1 min; 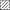, 3 min) and 1 h of post treatment storage time. ND, Non-detectable (below detection limit of 1.0 Log_10_ CFU/ml). Vertical bars indicate standard deviation. Column with different letters indicate a significant difference between bacterial strains and treatment time (*p* < 0.05).

Figure 3 shows inactivation patterns for *L. monocytogenes* EGD-e and knockout mutants after ACP treatment and 1 h PTST. All mutants were similarly affected by direct exposure to cold plasma for 1 min (*p* > 0.05), with different mutant responses noted only after 3 min cold plasma exposure. Δ*sigB* and Δ*lmo0799-C56A* were highly sensitive to ACP treatment, they exhibited lower survival levels than wild type and other mutant strains (*p* < 0.05). Δ*lmo0799-C56A* was reduced by 4.5 Log after 3 min exposure while Δ*sigB* mutants were below the detection limit. Both EGD-e WT and other mutant strains showed 2.5–2.7 log reduction after 3 min ACP treatment. No significant difference was recorded between the ACP inactivation rate of mutants (Δ*gadD1*, Δ*gadD2*, Δ*gadD3*, Δ*gadD2D3*, Δ*rsbR*, and Δ*lmo0799* mutants) and wild type parent strain. All strains tested were at the limit of detection (< 1.0 Log_10_ CFU/ml) after 5 min of treatment.

### Assessing Stress Responses to ACP Using *L. monocytogenes* Knockout Mutants

**FIGURE 4:**
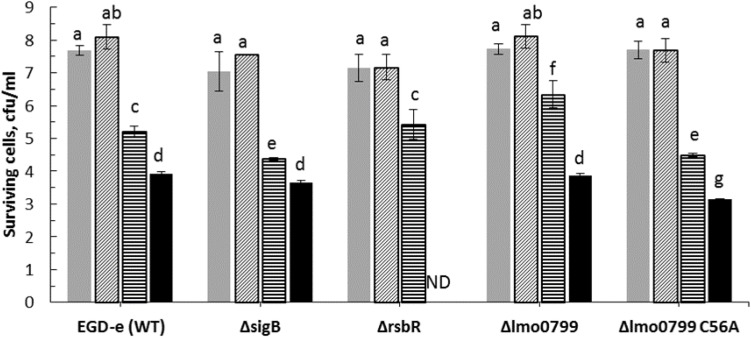
Influence of cold stress (4°C) on ACP inactivation efficacy for *L. monocytogenes* EGD-e (WT) and its mutant strains (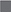, untreated control; 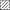, cultures exposed to cold stress for 1 h; 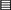, 1 min ACP; 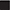, 1 min ACP to cultures exposed to cold stress). Experimental conditions: 1 min of direct plasma treatment at 80 kV following 1 h of Post treatment storage time. SD, standard deviation; ND, Non-detectable. Limit of detection was 1.0 Log_10_ CFU/ml.

**FIGURE 5:**
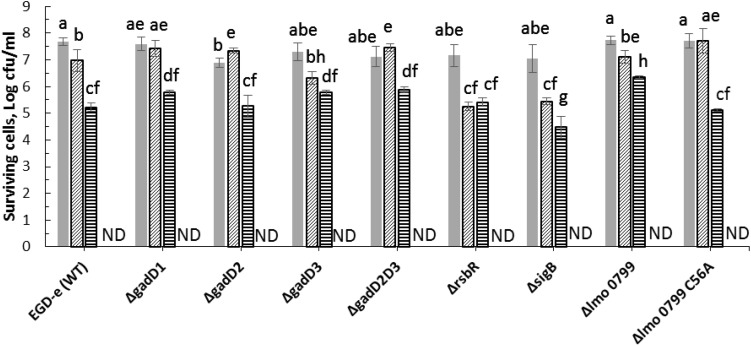
Effect of pre-exposure to mild acid conditions (pH 4) on ACP inactivation efficacy for *L. monocytogenes* EGD-e (WT) and mutant strains (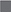, untreated control; 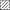, cultures exposed to acid stress for 1 h; 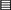 1 min ACP; 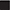 1 min ACP to cultures exposed to acid stress). Experimental conditions: 1 min of direct plasma treatment at 80 kV following 1 h of Post treatment storage time. SD, standard deviation; ND, non-detectable. Limit of detection was 1.0 Log_10_ CFU/ml.

In order to ascertain if sub-lethal stress exposure could influence *L. monocytogenes* behavior, ACP resistance was evaluated for the cultures exposed to cold (4°C) or acid (pH 4) stress for 1 h. Experiments focused on investigating the influence of stress adaptation on *L. monocytogenes* ACP resistance. The survival of *L. monocytogenes* EGD-e wild-type strain was compared to its knockout mutants following exposure to sub-lethal stress and ACP treatment (Figures 4, 5). Application of direct cold plasma was found to be effective for reduction of both *L. monocytogenes* EGD-e wild type and mutants. However, there were significant effects of bacterial pre-treatment and conditions observed.

Applying a mild cold stress (4°C) to *L. monocytogenes* EGD-e (WT) and knockout mutants (Δ*sigB*, Δ*lmo0799*, Δ*lmo0799-C56A*, and Δ*rsbR*) enhanced effects of ACP treatment. Notably, there was no significant difference observed between WT and mutant bacterial inactivation efficacy when exposed to cold stress or ACP treatment, except for the Δ*rsbR* mutant which was reduced below detection limit after 1 min of treatment. The limit of detection was 1.0 Log_10_ CFU/ml.

Adaptation of *L. monocytogenes* to stress can protect the pathogen to a variety of normal lethal conditions found in the environment (Lou and Yousef, 1997). In this study, all mutants subjected to acid pH 4.0 stress were susceptible to ACP treatment (Figure 5). All *L. monocytogenes* strains exposed to acid stress were reduced below detection limit immediately after 1 min of treatment.

### Effects of ACP on *L. monocytogenes* and *prfA* Mutant Biofilms

**FIGURE 6:**
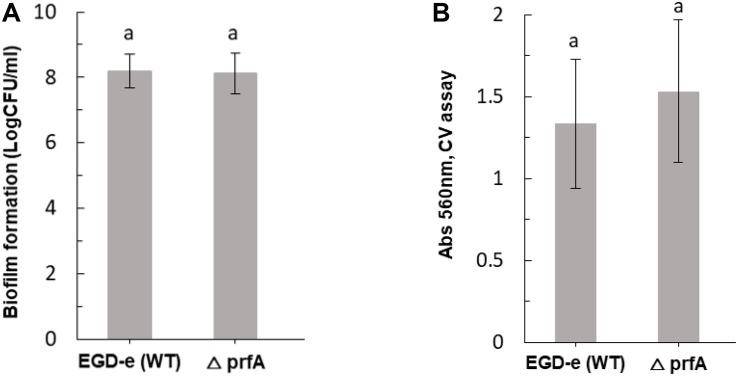
Impact of *prfA* on biofilm formation. Biofilm formation by *L. monocytogenes* EGD-e (WT) and △prfA mutant at 37^°^C in TSB for 48 h, quantified by **(A)** plate count and **(B)** crystal violet assay. Vertical bars indicate standard deviation. Column with different letters indicate a significant difference between EGD-e (WT) and prfA (*p* < 0.05).

Previous studies with *L. monocytogenes* reference strains 10403S and EGD revealed a significant requirement of *prfA* for biofilm formation (Lemon et al., 2010; Travier et al., 2013). Therefore, to investigate the role of *PrfA* in biofilm formation, Δ*prfA* mutant with in-frame deletion of *prfA* gene was analyzed for biofilm formation on 96 well microtiter plates using crystal violet and plate count assay (Figure 6). Both strains showed ability to attach to polystyrene 96-well microtiter plate. The Δ*prfA* mutant displayed similar levels of biofilm compared to wild type with no significant difference observed in means (*p* > 0.05). Deletion of *prfA* did not show any impact on growth and/or biofilm formation in Δ*prfA* mutant.

**FIGURE 7:**
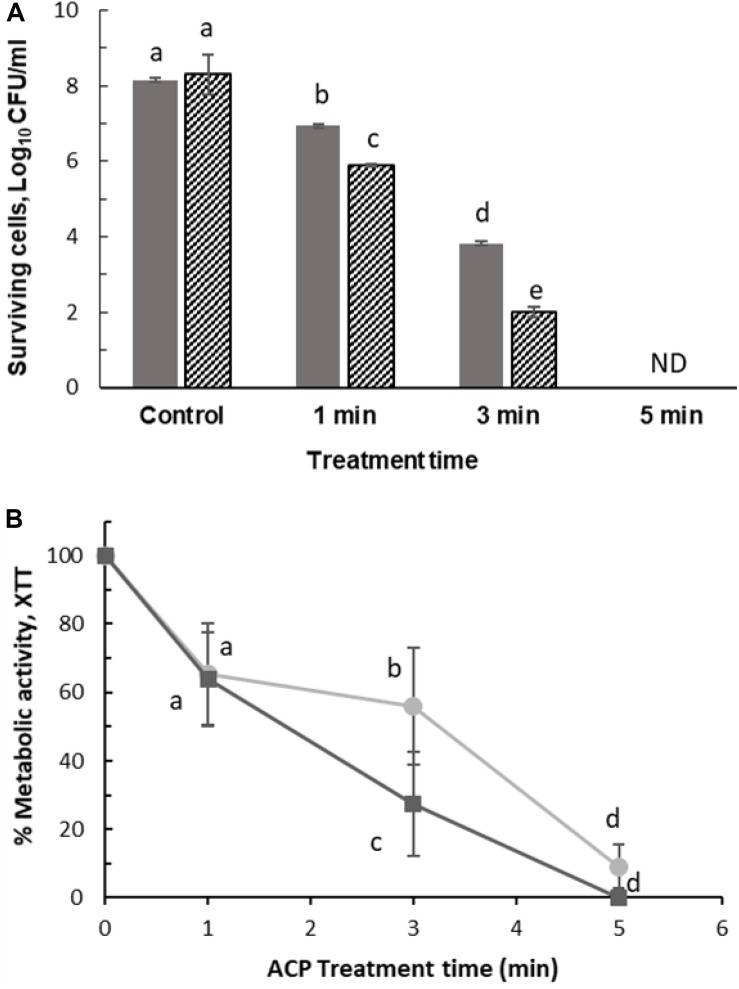
Surviving population of 48 h *L. monocytogenes* after ACP treatment for 1, 3, and 5 min following 1 h PTST. (A) Plate count: (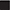) EGD-e WT, (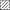) △*prfA*. ND, non-detectable (Limit of detection 1 Log_10_ CFU/ml). (B) XTT assay: (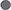) EGD-e WT assessed by XTT assay, (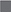) △*prfA* assessed by XTT assay. Vertical bars represent standard deviation. Column with different letters indicate a significant difference between bacterial strains and treatment time (*p* < 0.05).

Further to determine the relative contribution of the *prfA* gene, biofilms of the wild type and the Δ*prfA* mutant were treated with ACP for 1–5 min. Figure 7 shows that the mutant was significantly more sensitive to ACP treatment. ACP treatment for 1 min with 1 h PTST led to 1.2 log reduction (0.05 SD) of wild type biofilm compared to 2.4 log reduction (0.04 SD) for Δ*prfA* mutant. However, metabolic activity of wild type and *prfA* mutant were similarly affected by plasma for 1 min (*p* > 0.05), with difference in activity noted after 3 min plasma exposure. Figure 7B represents the effect of ACP treatment on viability and metabolic activity of the *L. monocytogenes* EGD-e and Δ*prfA* 48h biofilm which was analyzed by XTT assay.

### Effect of ACP on Gene Expression of Stress Induced Genes

**FIGURE 8:**
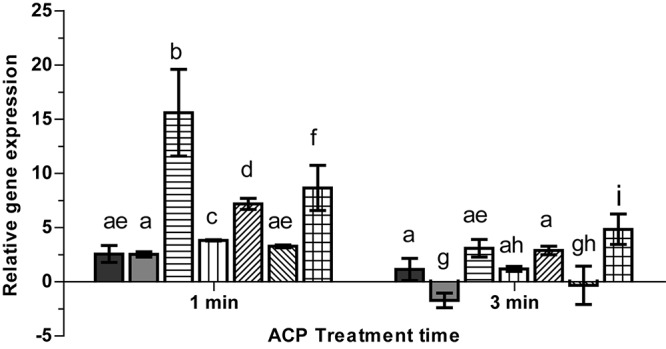
Effect of ACP on gene expression of stress related genes in L. monocytogenes EGD-e (WT). 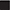, *rsbR;* 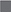, *prfA;* 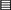, *sigB*; 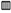, *gadD1*; 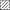, *gadD2*; 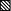, *gadD3*; 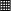, *lmo0799*. The *y*-axis represents the Log2 fold change between untreated cells and ACP treatment cells (for 1 min or 5 min), determined by the delta-delta C_T_ method. Vertical bars represent standard deviation in the mean of two independent repeats. Column with different letters indicate a significant difference between bacterial strains and treatment time (*p* < 0.05).

Atmospheric cold plasma has demonstrated an inhibitory effect on survival and at longer plasma exposure appears to have the capacity to kill *L. monocytogenes* cells (section “ACP Inactivation Efficacy Associated With Treatment Parameters”). Due to the inhibitory effects induced by extended in-package ACP exposure (5 min) to *L. monocytogenes* cells, the study focused on the biological effects induced by short term (1 and 3 min) in-package ACP exposures to allow detection of early effects in terms of ACP modulated gene activities. The qRT-PCR assay was optimized by determining primer annealing efficiency for each primer pair. The melting curve analysis for selected primer pairs showed single peaks confirming that there was no DNA contamination. The PCR efficiency for the primers ranged from 94 to 96%. To determine the specific contributions of the transcriptional regulator σ^B^ to expression of *L. monocytogenes* virulence and stress response genes, we measured transcript levels of selected σ^B^-dependent genes under sub-lethal stress conditions. Expression of target genes (*gadD1*, *gadD2*, *gadD3*, *sigB*, *prfA*, *rsbR*, and *lmo0799*) relative to expression of the 16S rRNA gene after 1 and 3 min exposure to ACP is presented in Figure 8. The result showed that exposure to sub-lethal 1 min ACP increased gene expression of stress associated genes. *SigB* showed highest gene expression, increasing by 15.60 ± 3.9 fold, followed by *gadD2* (7.19 ± 0.5) and *lmo0799* (8.6 ± 2.08) after 1 min exposure, compared to untreated controls. As expected when subjected to a harsh environment, *L. monocytogenes* increases expression levels of several stress related genes. However, longer ACP exposure decreased or in some cases down-regulated the gene expression. *prfA* was down-regulated 1.7 ± 0.6 fold after 3 min ACP while no significant induction or repression was noted for *gadD3*. The change in gene expression post treatment was mainly associated to ACP activity on bacteria.

## Discussion

*Listeria monocytogenes* is a persistent strain prevalent in food-borne illnesses, which is capable of surviving varied environmental and process conditions (NicAogáin and O’Byrne, 2016). Environmental stress such as acid, oxidative, cold or heat shock induced mutations can increase tolerance or resistance in bacteria (Delaquis and Bach, 2012). Bacterial adaptive strategies to stress, cause changes in the gene expression pattern to maintain viability under harsh conditions. In order to design efficient ACP treatments, it is essential to understand the behavior of the microorganism when exposed to different environmental and process stress conditions. Stress responses in *L. monocytogenes* have been extensively reported but investigations pertaining to cellular responses to cold plasma are still limited. ACP is increasingly explored as a tool for decontamination in a wide range of sectors (Patange et al., 2018; Sarangapani et al., 2018). In the present work, the efficiency of the ACP for inactivation of *L. monocytogenes* with the effects of commonly encountered process conditions as extrinsic conditions with mechanistic insights were examined using a series of mutants with deletions relevant to ACP and other environmental stresses.

Any effective antimicrobial agent must either penetrate or disrupt the bacterial membrane layer. Atmospheric cold plasma is an excellent source of reactive species which contribute to bacterial inactivation process (Arjunan et al., 2012; Wu et al., 2012) which occurs through bacterial membrane disruption via membrane disintegration and generation of ROS species. In this study, we confirm the enhanced level of intracellular ROS in L. monocytogenes strains after ACP treatment using DCFH-DA assay. The results from this assay suggest a highly oxidative stress environment inside *L. monocytogenes* cells in response to ACP treatment. Oxidative stress is well associated with deleterious effects on cells and intracellular components such as nucleic acids, lipids and proteins (Imlay, 2013). Previous studies have also suggested the link between bacteria inactivation by ACP and ROS production (Han et al., 2016a). ROS such as peroxide and superoxide generated inside a cell, cause a series of oxidation-reduction reactions mediated by iron-sulfur clusters, flavoproteins and the Fenton reaction, generating further very short-lived but high amounts of hydroxyl radical species which subsequently cause intracellular damage (Kohanski et al., 2007). Han et al. (2016b) demonstrated ACP treatment increased the overall amount of reactive species in *Escherichia coli* BW 25113 and its isogenic knockout mutants (*rpoS, soxR, soxS, oxyR*, and *dnaK* genes) that led to the increased cell membrane permeability and sensitivity with 1 and 24 h post treatment storage time.

The general mechanism by which cold plasma treatment can induce cell damage and cause cell death has been reported but there is scant information about the effects of ACP stress regulated genes and the protection or sensitivity against cold plasma treatment (Van Impe et al., 2018). The survival of *L. monocytogenes* EGD-e wild-type strain was compared with that of its isogenic mutants following exposure to cold plasma treatment. The same intensity cold plasma treatment had different effects on mutants with deleted genes associated with stress responses. The *sigB* mutant was more sensitive to ACP treatment than wild type and other mutants, with a higher level of ROS detected in these cells. SigB is responsible for regulation of several stress associated genes. SigB and sigB-dependent genes play an important role in oxidative stress, cold, and high hydrostatic pressure, etc. (Chaturongakul et al., 2008). SigB has demonstrated a protective role to oxidative stress (Ferreira et al., 2001). Therefore, in the absence of sigB, the bacterial strain was sensitive and readily inactivated by plasma treatment. To investigate the role of SigB in *L. monocytogenes* stress adaptation, we compared the survival of all of the strains under environmental stresses commonly encountered in food industry followed by sequential treatment with ACP technology. Different survival was observed between acid exposed wild type and sigB, however, with plasma treatment, both strains were inactivated within 1 min of treatment. This is consistent with other mutant strains; removing sigB and other sigB co-regulated stressosome gene had no effect on the physiological outcome of stress cross-protection. These results suggest that both acid and cold stress do not confer cross protection against ACP treatment. Despite the well-established role for SigB, there are several discrepancies reported by other researchers regarding its role under oxidative stress in *L. monocytogenes*. Studies by Boura et al. (2016) have shown that cells without SigB were more resistant to H_2_O_2_ by at least seven logs compared to its isogenic WT. The stationary phase cells grown aerobically were more resistant against H_2_O_2_ at 30^°^C than 37^°^C. In contrast, survival experiments by Chaturongakul and Boor (2004) showed considerably reduced survival of the Δ*sigB* strain relative to the parent strain and indicated a role for σ^B^ in oxidative-stress resistance in *L. monocytogenes* 10403S during exposure to cumene hydroperoxide (CHP). Interestingly in this study, hypersensitivity was observed in cells pre-adapted to cold or low pH conditions.

*Listeria monocytogenes* utilizes the glutamate decarboxylase (GAD) system to maintain pH homeostasis within the cell and survive under acidic conditions. Three genes GadD1, GadD2, and GadD3 are critical for encoding glutamate decarboxylases, which with two antiporters (gadT1 and gadT2) play an important role in *L. monocytogenes* survival and facilitates growth at acidic pH conditions (Wemekamp-Kamphuis et al., 2004; Cotter et al., 2005). Feehily et al. (2014) demonstrated that the deletion of *gadD3* together with either *gadD1* or *gadD2*, reduced acid tolerance in *L. monocytogenes* EGD-m. However, resistant single mutants (*gadD1* and *gadD2*) showed greater acid survival than the wild type strain. Deletion of the Gad genes (*gadD1*, *gadD2*, and *gadD3*) seemed to confer no protection against mild acid or ACP derived oxidative stress. Along with the GAD system, other genes such as *rsbR, sigB, lmo0799, and lmo0799-C56A* in *L. monocytogenes* are known to be responsible to protect the bacterium from multiple stress conditions. Therefore, the survival of wild type and *rsbR, sigB, lmo799*, and *lmo799-C56A* gene mutant strains were tested under acid stress conditions. The loss of sigma B or sigma activating protein rsbR and lmo0799 conferred no significant difference to strain survival at low pH; indicating that σ^B^ is not solely responsible for the acid tolerance response. Pre-adaptation to sub-lethal pH (4.0) and cold (4^°^C) did not enhance the survival of either strain following exposure to ACP treatment. All pre-adapted *L. monocytogenes* strains were highly susceptible and were reduced below the detection limit within 1 min of the ACP treatment. The stress conditions used in this study may have been sufficiently lethal to have overwhelmed the possible *sigB* contributions to cellular survival.

RT-PCR was applied for gene expression studies, focused on expression changes of stress associated genes of *L. monocytogenes* (wild-type bacteria) after sub-lethal exposure to ACP. Interestingly, an increase in expression was recorded for all stress-associated genes, particularly *sigB*, analyzed after 1 min of ACP treatment. The RT-PCR results show a significant association between ACP exposure and *sigB* expression, confirming its essential function for the increased tolerance to stress factors. This highlights the importance of optimizing treatment parameters used to control pathogenic bacteria like *L. monocytogenes*, which under sub-lethal treatments could lead to non-compliance or resistance to any treatment. Further studies are necessary to elucidate whether these differences affect phenotypic responses of the bacteria. A significant correlation was observed for the expression of *sigB* associated genes and *sigB* after ACP treatment in *L. monocytogenes*. The *sigB* overexpression mainly occurred at sub-lethal ACP exposure (1 min) with higher gene expression of *rsbR* and *lmo0799*. SigB activation by stress is a complex process which has extensively been studied in *B. subtilis* (Hecker et al., 2007). RsbR and its paralogs (RsbS and RsbT) are thought to sense stress signals with protruding N-termini present. This signal is then transduced into core stressosome which ultimately leads to activation of SigB. While this is yet to be confirmed in *L. monocytogenes*, a similar process is hypothesized. RsbR and its paralogs such as lmo0799 in *L. monocytogenes* are integral parts of the stressosome complex, its contribution for σ^B^ activation cascade and σ^B^ activity (reviewed in Van der Steen et al., 2012; O’Donoghue et al., 2016) consequently leads to the transcription of the general stress response (GSR) regulon (NicAogáin and O’Byrne, 2016). In *L. monocytogenes* EGD-e exposed to ACP for 3 min, the transcriptomic analysis revealed reduced expression of *sigB* and other stress associated genes. Hence, *L. monocytogenes* shows changes in its resistance to different durations of plasma treatment due to gene alterations.

The intensity of ACP-induced oxidative stress generates high concentrations of intracellular ROS/RNS species. These reactive species react with nearby organics leading to chain oxidation and destruction of DNA molecules as well as cellular membranes and other cell components (Dobrynin et al., 2009). The deoxyribose sugar and the nucleobases of DNA are readily susceptible to direct oxidative/nitrosative attacks by ROS/RNS (Arjunan et al., 2015). Following exposure to ACP, the SOS response regulon (consisting of several genes responsible for DNA repair mechanism, cell division) are significantly up-regulated (Sharma et al., 2009). Previous studies by Xu et al. (2015), demonstrated increase in Nfo gene (encodes for endonuclease IV, a DNA repair protein), indicating that the bacterial DNA structures are gradually damaged with the increase in plasma exposure. The DNA repair protection largely depends upon the type, quantity and the exposure time of bacterial cells to generated ROS. In addition to oxidation of the deoxyribose sugar, ROS/RNS directly damage DNA damage repair enzymes and polymerases (Arjunan et al., 2015) thus slowing the repair processes or preventing replication altogether. Similarly, Sharma et al. (2009) suggests although plasma treatment leads to the induction of uvrA and uvrB (that detect damaged nucleotides), the absence of uvrC, uvrD, and polA leads to incomplete induction of DNA damage repair mechanisms, thus causing cellular damage.

The σ^B^ regulon is the largest stress response regulon in *L. monocytogenes* and overlaps with several other regulatory systems. Environmental stress conditions can prime σ^B^-regulated virulence functions of *L. monocytogenes*, i.e., σ^B^ also contributes to transcription of the gene encoding the global *L. monocytogenes* virulence gene regulator, Positive regulatory factor A (*PrfA*) (reviewed in Scortti et al., 2007; O’Byrne and Karatzas, 2008); binds to the specific DNA sequence known as *prfA* box and positively regulates the expression of *L. monocytogenes* virulence genes including itself. Luo et al. (2013) and Lemon et al. (2010) highlighted the importance of *prfA* gene leading on extracellular biofilm formation and virulence; and elimination of *prfA* gene led to reduced biofilm formation with altered gene expression patterns observed in several different strains of *L. monocytogenes*. In this study, both *L. monocytogenes* EGD-e (WT) and △*prfA* strains showed similar biofilm formation in TSB at 37°C. Such data suggest that, besides prfA, additional determinants could be responsible for development and formation of biofilms. While similar biofilm formation was observed in both strains, Δ*prfA* was significantly more sensitive than the wild type strain, with 6.3 ± 0.02 Log_10_ CFU/ml reduction of culturable cells and 72% reduction in metabolically active cells after 3 min of treatment. Further, the gene expression studies revealed that sub-lethal treatment of 1 min induced *prfA* gene expression; however, prolonged treatment of 3 min repressed the *prfA* expression by 1.7 ± 0.6 fold. Expression of *prfA* gene is highly regulated by multiple promoters; it can be transcribed as bicistron, together with the upstream plcA gene and monocistronically from two promoters (P1*prfA* and P2*prfA*) directly upstream from the *prfA*, one of which is under control of *sigB* (Scortti et al., 2007). The partially σ^B^-dependent P2*prfA* promoter contributes to the majority of *prfA* transcript levels in both intra- and extracellular bacteria (Chaturongakul et al., 2008). Therefore, when bacteria were exposed to ACP exposure, *sigB* was highly expressed which may have contributed to increased gene expression of *prfA* (Rauch et al., 2005). Similarly, Xu et al. (2015) demonstrated, plasma exposure up-regulated genes (IcaA, SarA, *sigB*, Rbf, LuxS) responsible for polysaccharide intercellular adhesion, regulation and biofilm formation in *Staphylococcal* biofilms and significant difference in gene sensitivity was observed with plasma exposure for 10 and 30 min. Xu et al. (2015) employed a different cold plasma device, however, the key effectors are likely to be similar. Consequently, bacteria such as *L. monocytogenes* and *S. aureus* respond to sub-lethal cold plasma-derived stress conditions to regulate biofilm formation via bio-molecular processes on the genetic level. Hence, it is suggested that bacteria may show potential changes in their resistance to cold plasma treatment time due to the sensitivity to the cold plasma-driven environmental alteration and their impact on DNA repair mechanisms.

## Conclusion

In summary, this study underlines and confirms that ACP treatment effectively reduced *L. monocytogenes* and its mutants with 3–5 min of treatment under stressed/non-stressed/biofilm conditions. The effect of different durations of ACP treatment was analyzed for *L. monocytogenes* EGD-e at the gene transcription level. The results indicate *sigB* is important for general stress resistance, loss of *sigB* gene significantly reduced bacterial resistance to ACP treatment. In addition, *sigB* gene showed the highest gene expression under sub-lethal ACP treatment. The results show that cold plasma exposure can induce gene expression to a different degree. However, whether cold plasma exposure facilitates or attenuates resistance due to sub-lethal treatments requires further investigation. Hence, an improved understanding of L. monocytogenes response to different stress factors is necessary, which may help reveal comprehensive mechanisms of plasma induced effects. Further studies could evaluate the extent of changes in virulence or other biological properties as a response to stress and resistance-associated gene expression. This would contribute to an understanding of how to control and reduce these events. The experimental results presented here provide a baseline for understanding the bacterial genetic response to cold plasma stress and offers promising insights for optimizing ACP applications.

## Data Availability Statement

Data can be found here: https://arrow.dit.ie/datas/8.

## Author Contributions

PB, AP, PC, CO’B, KK, and DB conceived and designed the experiments. AP performed the experiments. PB and AP wrote the manuscript.

## Conflict of Interest

The authors declare that the research was conducted in the absence of any commercial or financial relationships that could be construed as a potential conflict of interest.
