# Supplementary material for: The Effect of Atmospheric Cold Plasma on Bacterial Stress Responses and Virulence Using Listeria monocytogenes Knockout Mutants
Source: Front Microbiol. 2019 Dec 11;10:2841. doi: 10.3389/fmicb.2019.02841 (PMC6918802; doi:10.3389/fmicb.2019.02841)
Supplement: Supplementary file 1 [file Table_1.docx]

Supplementary Material

The effect of atmospheric cold plasma on bacterial stress responses and virulence using *Listeria monocytogenes* knockout mutants

**Apurva Patange^1^, Conor O’ Byrne^2^, Daniela Boehm^1^, P.J. Cullen^1,3^ Kevin Keener ^1,4^ and Paula Bourke^1,5*^**

^1^School of Food Science and Environmental Health, Technological University Dublin, Ireland

^2^Bacterial Stress Response Group, School of Natural Sciences, National University of Ireland Galway, Ireland

^3^ School of Chemical and Biomolecular Engineering, University of Sydney, Australia

^4^ Department of Food Science and Human Nutrition, Iowa State University, United States

^5^ School of Biological Sciences, Queens University Belfast, Northern Ireland, United Kingdom

* Correspondence: [paula.bourke@dit.ie](mailto:paula.bourke@dit.ie)

# Supplementary Data

**Table S1:** Primers used in this study

| **Description** | **Sequence (5’-3’)** | **Annealing temperature** | **References** |
| --- | --- | --- | --- |
| FOR lmo0799 | ACAAATGTAGCCGCCCTTC | 58°C | O'Donoghue et al., 2016 |
| REV lmo0799 | CATCTCGCAACCTCTACCTC |  |  |
| FOR gadD1 | ACAAATACGCCACGCATC | 58°C | Feehily et al., 2014 |
| REV gadD1 | GGCAAGAACCATAAGAATCCAC |  |  |
| FOR gadD2 | TCATTCCTAACTGCCATTTCC | 58°C | Feehily et al., 2014 |
| REV gadD2 | TGGAATGAGAATAGTGGACGG |  |  |
| FOR gadD3 | GAACCTCCTTATAAGTACCATC | 54°C | Feehily et al., 2014 |
| REV gadD3 | GGTGGTTACGGTGCATTC |  |  |
| FOR sigB | CTATATTGGATTGCCGCTTAC | 54°C | Utratna et al., 2011 |
| REV sigB | CAAACGTTGCATCATATCTTC |  |  |
| FOR 16S rRNA | TGGGGAGCAAACAGGATTAG | 58°C | Utratna et al., 2011 |
| REV 16S rRNA | TAAGGTTCTTCGCGTTGCTT |  |  |
| FOR rsbR | GCAAACCGCACAATAAGAGAG | 58°C | O'Donoghue et al., 2016 |
| REV rsbR | GCTCCGCTAAACGTAATTCC |  |  |
| FOR prfA | ACGGGAAGCTTGGCTCTATT | 54°C | Ondrusch and Kreft., 2011 |
| REV prfA | TGCGATGCCACTTGAATATC |  |  |
